# Proteomic remodeling of outer membrane vesicles in antibiotic-resistant *Helicobacter pylori* reveals adaptive stress responses

**DOI:** 10.3389/fmicb.2026.1944144

**Published:** 2026-09-04

**Authors:** Tuana Nur Aydınlar, Sümeyye Akçelik-Deveci, Meltem Ayaş, Sinem Öktem-Okullu

**Affiliations:** 1 Department of Medical Microbiology, Institute of Health and Science, Acibadem Mehmet Ali Aydinlar University, Atasehir, Istanbul, Türkiye; 2 Department of Molecular Biology and Genetics, Faculty of Sciences, Boğaziçi University, Istanbul, Türkiye; 3 Department of Medical Laboratory Techniques, Vocational School of Health Services, Acibadem Mehmet Ali Aydinlar University, Istanbul, Türkiye; 4 Department of Medical Microbiology, School of Medicine, Acibadem Mehmet Ali Aydinlar University, Atasehir, Istanbul, Türkiye

**Keywords:** adaptive stress responses, *Helicobacter pylori*, OMV, outer membrane vesicles in antibiotic-resistance, proteomic data

## Abstract

**Background:**

Antibiotic resistance in *Helicobacter pylori* has become a major obstacle to successful eradication therapy worldwide. Although outer membrane vesicles (OMVs) are increasingly recognized as key mediators of bacterial adaptation and host interaction, their contribution to antibiotic resistance in *H. pylori* remains poorly understood.

**Methods:**

Metronidazole and levofloxacin-resistant derivatives of the *H. pylori* G27 strain were generated by stepwise antibiotic exposure. OMVs isolated from susceptible and resistant strains were characterized by transmission electron microscopy and dynamic light scattering, followed by comparative LC–MS/MS analysis of whole-cell and OMV proteomes. Functional annotation and enrichment analyses were performed to identify resistance-associated biological processes.

**Results:**

Comparative proteomic analysis revealed remodeling of both cellular and OMV-associated protein profiles following acquisition of antibiotic resistance. While 236 proteins were shared among all strains, distinct resistance-associated protein signatures were identified in the levofloxacin- and metronidazole-resistant derivatives. Functional enrichment highlighted pathways associated with membrane transport, oxidative stress response, metabolic adaptation, and cellular envelope organization. OMV proteomes differed from their corresponding whole-cell proteomes, a pattern consistent with, though not proof of, selective protein packaging. Conserved vesicle-associated proteins, including UreA, UreB, GroEL, and GroES, were detected in OMVs from all strains, whereas OMVs from the levofloxacin-resistant derivative contained proteins associated with redox homeostasis and iron metabolism, including SelA and FeoB. In addition, differential representation of the virulence factors CagA and VacA was observed between susceptible and resistant OMVs.

**Conclusion:**

Acquisition of antibiotic resistance in *H. pylori* is accompanied by coordinated remodeling of both the cellular proteome and OMV cargo. The selective representation of stress adaptation, transport, and virulence associated proteins within OMVs is consistent with the hypothesis that selective protein packaging may contribute to bacterial adaptation under antibiotic pressure and identifies OMV-associated proteins and pathways as potential targets for future therapeutic intervention.

## Graphical Abstract

**Figure:**
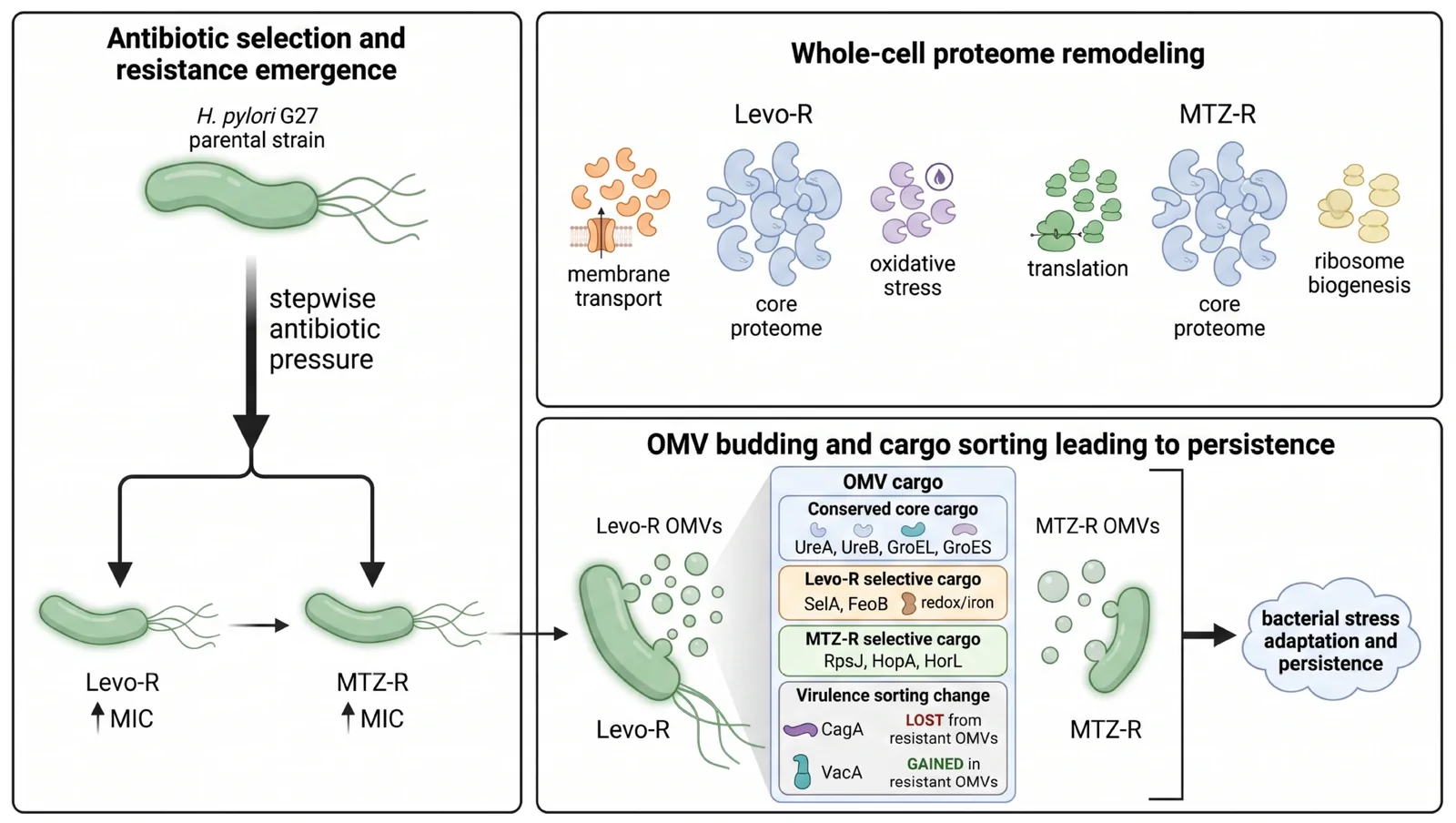
Created with BioRender.com.

## 1 Introduction

*Helicobacter pylori* infects nearly half of the world’s population and is a major cause of chronic gastritis, peptic ulcer disease, mucosa-associated lymphoid tissue (MALT) lymphoma, and gastric adenocarcinoma (Crowe, 2019; Hooi et al., 2017). Owing to its well-established role in gastric carcinogenesis, the World Health Organization has classified *H. pylori* as a Group I carcinogen (International Agency for Research on Cancer (IARC), 1994; Kusters et al., 2006). Although eradication therapy has substantially reduced the burden of *H. pylori*-associated diseases, treatment success has declined worldwide because of the increasing prevalence of antibiotic-resistant strains (Savoldi et al., 2018).

Current eradication regimens typically include clarithromycin, metronidazole, and fluoroquinolones such as levofloxacin as part of standard triple or quadruple therapy (Malfertheiner et al., 2017). However, global surveillance studies have reported increasing resistance rates to these antibiotics, particularly for clarithromycin and metronidazole, with regional prevalence frequently exceeding recommended therapeutic thresholds (De Francesco et al., 2010;

Hasanuzzaman et al., 2024). The molecular basis of antibiotic resistance in *H. pylori* has largely been attributed to mutations in antibiotic target genes, including the 23S rRNA gene for clarithromycin resistance, *rdxA*/*frxA* for metronidazole resistance, and *gyrA* for fluoroquinolone resistance (Debets-Ossenkopp et al., 1999; Tankovic et al., 2003; Versalovic et al., 1996). Although these well-established genetic mechanisms explain resistance to individual antibiotics, they do not fully account for the remarkable ability of *H. pylori* to survive prolonged antibiotic exposure. Increasing evidence suggests that additional mechanisms, including membrane remodeling, activation of stress-response pathways, and extracellular vesicle production, contribute to antibiotic tolerance and bacterial persistence (Windgassen et al., 2000).

Outer membrane vesicles (OMVs) are nanosized lipid bilayer vesicles naturally released from the outer membrane of Gram-negative bacteria (Kulp and Kuehn, 2010). They contain lipopolysaccharides, outer membrane proteins, periplasmic enzymes, nucleic acids, and virulence factors, and function as important mediators of bacterial communication, immune modulation, horizontal gene transfer, and interactions with the host (Li et al., 2024; Schwechheimer and Kuehn, 2015). In recent years, OMVs have also emerged as key components of bacterial stress responses. Exposure to antibiotics, oxidative stress, and envelope damage has been shown to stimulate OMV production in several Gram-negative bacteria, suggesting that vesiculation represents an important physiological response that enhances bacterial fitness under adverse environmental conditions (Lu et al., 2025; Toyofuku et al., 2019).

Several mechanisms have been proposed to explain the contribution of OMVs to antibiotic resistance. Vesicles can sequester antimicrobial compounds, thereby reducing the intracellular concentration of antibiotics. They may also transport resistance-associated proteins, such as β-lactamases, and facilitate the dissemination of resistance determinants within bacterial communities (Ciofu et al., 2000; Rumbo et al., 2011; Schaar et al., 2011). In addition, accumulating evidence indicates that OMV cargo is selectively regulated rather than randomly packaged. Alterations in vesicle protein composition following antibiotic exposure suggest that OMVs participate in coordinated bacterial responses to environmental stress by enriching proteins involved in survival and cellular homeostasis (Orench-Rivera and Kuehn, 2016).

In *H. pylori*, OMVs have been investigated primarily for their roles in virulence and host-pathogen interactions. Vesicles derived from this pathogen deliver key virulence factors, including CagA, VacA, and other immunomodulatory proteins, to gastric epithelial cells, thereby contributing to epithelial injury and modulation of host immune responses (Olofsson et al., 2010; Parker et al., 2010; Jarzab et al., 2020). Although several studies have characterized the protein composition and virulence-associated cargo of *H. pylori* OMVs, little is known about how antibiotic resistance influences vesicle composition or whether resistant strains selectively package proteins that promote stress tolerance, metabolic adaptation, and long-term bacterial persistence.

In the present study, we compared the whole-cell and OMV proteomes of antibiotic-susceptible and experimentally generated metronidazole- and levofloxacin-resistant *H. pylori* G27 strains using LC–MS/MS-based proteomics. We hypothesized that prolonged antibiotic exposure would induce selective remodeling of OMV protein cargo, leading to enrichment of proteins involved in stress responses, membrane integrity, redox homeostasis, and metabolic adaptation. By integrating comparative proteomics with functional enrichment and protein–protein interaction analyses, we sought to identify OMV-associated proteins and biological pathways that may contribute to bacterial persistence under antibiotic pressure and provide new insights into the role of OMVs in antibiotic resistance.

## 2 Materials and methods

### 2.1 Bacterial strains and culture conditions

The reference *Helicobacter pylori* G27 strain and its experimentally generated antibiotic-resistant derivatives were recovered from frozen stocks and cultured on Mueller–Hinton agar supplemented with 5% defibrinated sheep blood. Plates were incubated at 37°C for 3–4 days under microaerophilic conditions (approximately 5% O2, 10% CO2, and 85% N2) using a commercial gas-generating system.

For liquid cultures, bacterial colonies were inoculated into Brucella broth supplemented with 10% fetal bovine serum (FBS) and incubated at 37°C under microaerophilic conditions with shaking at 180 rpm for 72 h. Culture purity, bacterial morphology, and motility were routinely monitored by light microscopy.

### 2.2 Antimicrobial susceptibility testing

Minimum inhibitory concentrations (MICs) of metronidazole and levofloxacin were determined using the agar dilution method according to the recommendations of the European Committee on Antimicrobial Susceptibility Testing (EUCAST) (European Committee on Antimicrobial Susceptibility Testing (Eucast), 2024; Malfertheiner et al., 2017).

Mueller–Hinton agar supplemented with 5% defibrinated sheep blood was prepared with two-fold serial dilutions of metronidazole (0.25–32 μg/mL) or levofloxacin (0.031–4 μg/mL). Bacterial suspensions were adjusted to a 2.0 McFarland standard (approximately 1 × 10^7^ CFU/mL), and 3 μL aliquots were spotted onto antibiotic-containing agar plates. Following incubation under microaerophilic conditions at 37°C for 72 h, MIC values were defined as the lowest antibiotic concentrations that completely inhibited visible bacterial growth. All susceptibility assays were performed in triplicate.

### 2.3 Generation of antibiotic-resistant strains

Antibiotic-resistant derivatives were generated by serial passaging under progressively increasing concentrations of metronidazole or levofloxacin. Bacteria were initially cultured at the highest antibiotic concentration permitting visible growth and subcultured for five consecutive passages before transfer to the next concentration. At each step, a single colony was selected to maintain clonal populations. The stability of the resistant phenotype was confirmed by repeated MIC determination after several passages on antibiotic-free medium.

### 2.4 Genomic DNA ısolation and virulence gene detection

Genomic DNA was isolated from bacterial pellets using a commercial bacterial DNA extraction kit according to the manufacturer’s instructions. DNA concentration and purity were determined spectrophotometrically. Virulence-associated genes were detected by polymerase chain reaction (PCR) using gene-specific primers. Amplified products were separated on 1.5% agarose gels and visualized under ultraviolet illumination.

### 2.5 Isolation of outer membrane vesicles

Outer membrane vesicles (OMVs) were isolated from 3-day bacterial cultures grown under microaerophilic conditions. Resistant strains were cultured in the presence of the corresponding antibiotic at the previously established resistance concentration.

Bacterial cells were removed by centrifugation at 3,000 × g for 15 min at 4°C. The resulting supernatants were subsequently passed through sterile 0.22-μm pore-size membrane filters to remove residual bacterial cells and large debris prior to sequential ultracentrifugation. Filtered supernatants were then subjected to ultracentrifugation at 36,000 × g for 1 h followed by 200,000 × g for an additional 1 h at 4°C. The resulting OMV pellets were resuspended in sterile phosphate-buffered saline (PBS) and stored at −80°C until further analysis.

### 2.6 Characterization of outer membrane vesicles

#### 2.6.1 Dynamic light scattering

The hydrodynamic diameter and polydispersity index (PDI) of purified OMVs were determined by dynamic light scattering (DLS). Prior to analysis, vesicle suspensions were passed through 0.22-μm membrane filters to remove particulate aggregates. Measurements were performed at 25°C, and the mean hydrodynamic diameter (Z-average) together with the PDI was recorded.

#### 2.6.2 Transmission electron microscopy

OMV morphology was examined by transmission electron microscopy (TEM). Purified vesicles were fixed with 2.5% glutaraldehyde, pelleted by ultracentrifugation at 100,000 × *g* for 2 h at 4°C, post-fixed with osmium tetroxide, dehydrated through a graded ethanol series, and embedded in epoxy resin. Ultrathin sections were stained with uranyl acetate and lead citrate prior to microscopic examination.

### 2.7 Protein extraction, quantification, and SDS–PAGE

OMV proteins were extracted by lysing purified vesicles in RIPA buffer followed by incubation on ice for 30 min. Lysates were clarified by centrifugation at 16,000 × *g* for 10 min at 4°C, and the resulting supernatants were collected.

Whole-cell proteins were extracted from bacterial pellets using a detergent-based protein extraction reagent according to the manufacturer’s recommendations. Protein extracts were clarified by centrifugation and stored at −80°C until further analysis.

Protein concentrations were determined using the bicinchoninic acid (BCA) assay. Equal amounts of protein (10 μg per lane) were mixed with Laemmli sample buffer containing β-mercaptoethanol, heated at 95°C for 5 min, and separated by SDS–PAGE. Protein profiles were visualized using either Coomassie Brilliant Blue or silver staining.

### 2.8 LC–MS/MS proteomic analysis

Protein samples were subjected to in-gel digestion using sequencing-grade trypsin. Excised gel bands were washed with 50% acetonitrile in 25 mM ammonium bicarbonate, dehydrated, and incubated overnight at 37°C with trypsin. Peptides were extracted, dried by vacuum centrifugation, reconstituted in 0.1% formic acid, and analyzed by liquid chromatography coupled with tandem mass spectrometry (LC–MS/MS).

Peptide separation was performed using an Ultimate 3000 RSLC nano system (Dionex, Thermo Scientific, CA, United States) coupled to a Q Exactive mass spectrometer (Thermo Scientific, United States), controlled by Xcalibur 4.0 software (Thermo Fisher Scientific, CA, United States). Peptides were pre-concentrated and desalted on a trap column before separation on an Acclaim PepMap RSLC C18 analytical column (75 μm × 25 cm, 2 μm particle size, 100 Å pore size, Thermo Scientific, CA, United States), using mobile phases A (0.1% formic acid) and B (80% acetonitrile, 0.1% formic acid) at a flow rate of 300 nL/min over a 160-min gradient (6% B for 9 min, 6–22% B over 71 min, 22–44% B over 55 min, 44–90% B over 7 min, 90% B for 8 min, 90–6% B over 10 min, and 6% B for 5 min). Mass spectra were acquired in positive ion mode using data-dependent acquisition (DDA), in which the ten most abundant precursor ions were selected for fragmentation during each scan cycle. Full-scan MS1 spectra were acquired at a resolution of 70,000, over a scan range of 250–2000 m/z, with an automatic gain control (AGC) target of 3 × 10^6^ and a maximum injection time of 60 ms; spray voltage was set to 2.3 kV. MS/MS fragmentation was performed by higher-energy collisional dissociation (HCD) at a resolution of 17,500, with an AGC target of 1 × 10^6^, a maximum injection time of 100 ms, an isolation window of 2.0 m/z, and normalized collision energy (NCE) of 27. The instrument was calibrated prior to analysis using a standard positive-mode calibrant (LTQ Velos ESI Positive Ion Calibration Solution, Pierce, United States).

Raw mass spectrometry data were processed using Proteome Discoverer 2.2 software (Thermo Fisher Scientific, United States) and searched against the UniProt *H. pylori* G27 reference proteome database. Searches were performed with a peptide mass tolerance of 10 ppm, an MS/MS fragment mass tolerance of 0.2 Da, and a mass accuracy of 2 ppm, allowing up to one missed cleavage and a minimum peptide length of six amino acids. Carbamidomethylation of cysteine was set as a fixed modification, and oxidation of methionine and deamidation of asparagine were set as variable modifications. Protein identifications required a minimum of one identified peptide per protein and were accepted at a false discovery rate (FDR) threshold of <1%. For each identified protein, the number of peptide-spectrum matches (PSMs) and the number of unique peptides were extracted from the Proteome Discoverer output and are provided in Supplementary Table S_PSM to support the confidence of the protein identifications summarized in Table 1.

**TABLE 1:** Distribution of selected virulence, membrane, efflux, and stress-associated proteins in whole-cell and outer membrane vesicle (OMV) proteomes of parental and antibiotic-resistant *Helicobacter pylori* G27 strains.

Protein	Function	G27 Cell	G27 OMV	Levo Cell	Levo OMV	MTZ Cell	MTZ OMV
CagA	Virulence	+	+	−	−	+	−
VacA	Toxin	+	−	+	+	+	+
HefB	Efflux	−	−	+	−	+	−
SelA	Redox	+	−	+	+	+	−
HopA	Outer membrane	−	−	−	−	−	+
HorL	Outer membrane	−	−	+	−	−	+

Presence (+) or absence (−) of representative proteins identified by LC–MS/MS in whole-cell lysates and the corresponding OMV preparations of the parental G27 strain and its levofloxacin-resistant (Levo-R) and metronidazole-resistant (MTZ-R) derivatives. Peptide-spectrum match (PSM) and unique peptide counts supporting each protein identification are provided in Supplementary Table S_PSM.

### 2.9 Bioinformatics analysis

Identified proteins were functionally annotated using R-based bioinformatic workflows. GO biological process, molecular function, and cellular component enrichment analyses were performed using the clusterProfiler package in R for proteins detected in the parental *Helicobacter pylori* G27 strain and its antibiotic-resistant derivatives. Enriched terms were retained using FDR < 0.05 and a minimum of two matched proteins. Comparative enrichment patterns were visualized as bubble plots in R, where bubble size represented the number of proteins assigned to each term and color intensity indicated −log10(FDR).

Unique and shared proteins across experimental groups were identified using the jvenn online platform. A protein was classified as uniquely detected in a given group when it was identified with at least one peptide and accepted at a protein-level FDR < 1%, according to the LC–MS/MS identification criteria described above, and was absent from all other protein lists included in the same comparison. Proteins detected in more than one group were classified as shared. Venn diagrams were refined in Inkscape for publication. OMV-associated proteins were additionally mapped onto enriched GO terms derived from the corresponding whole-cell bacterial proteomic datasets, and these protein–GO term relationships were visualized using chord diagrams in R.

### 2.10 Statistical analysis

MIC determinations were performed in triplicate in three independent experiments. OMVs used for proteomic analyses were prepared from two independent biological replicates. Dynamic light scattering measurements were derived from single determinations and were used to characterize the OMV size distribution descriptively; no statistical comparison of vesicle size was performed. Because proteomic profiling was based on two independent biological replicates analyzed by data-dependent acquisition, comparisons between strains were treated as exploratory and interpreted qualitatively. Proteins were classified as detected or not detected rather than by quantitative label-free differential abundance, and the absence of a protein in a given dataset should therefore be interpreted with caution, as it may reflect the workflow’s detection limits rather than true biological absence. The graphical abstract was created using BioRender (BioRender, 2026).

## 3 Results

### 3.1 Generation and phenotypic confirmation of antibiotic-resistant *H. pylori* derivatives

**FIGURE 1:**
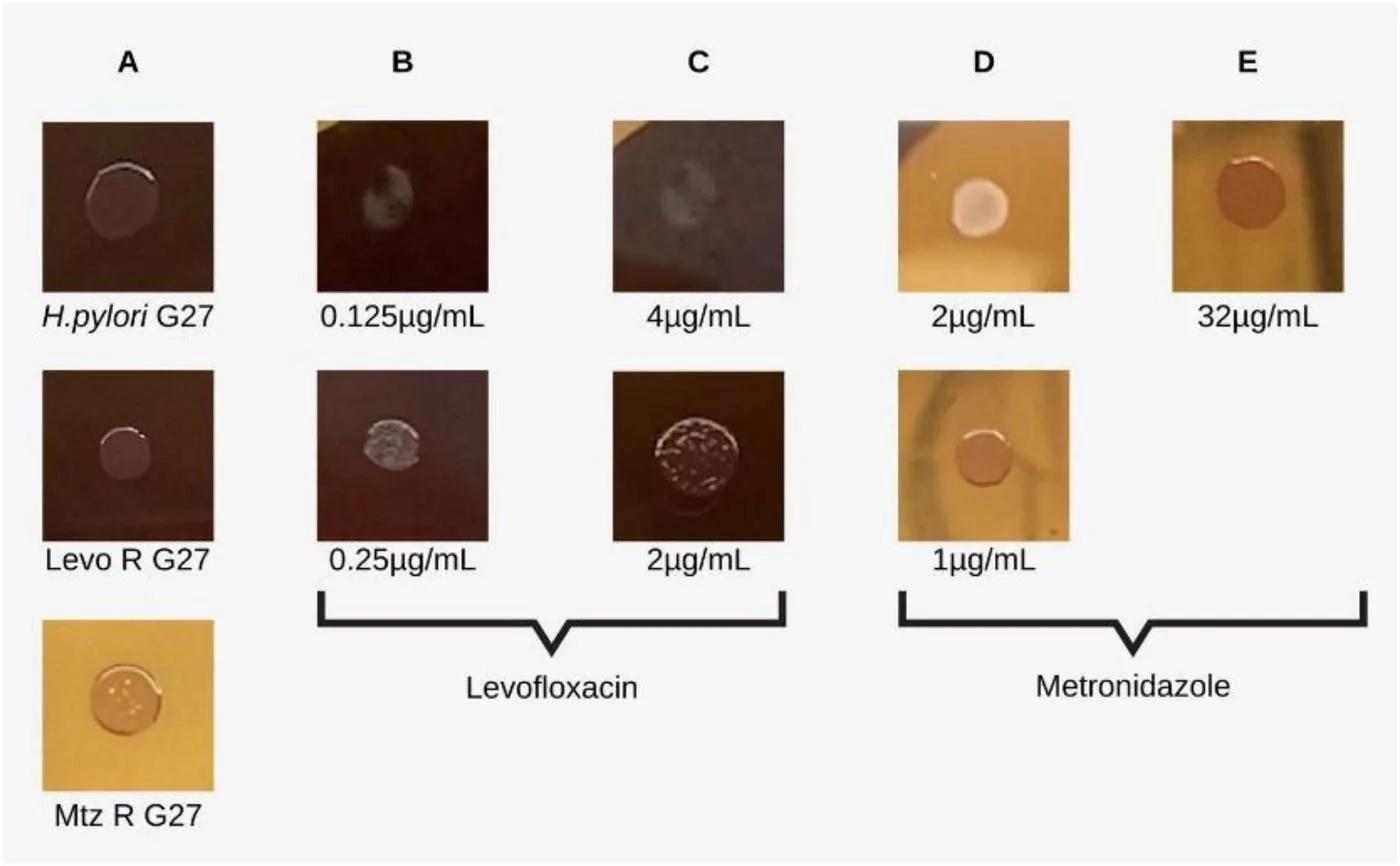
Phenotypic confirmation of antibiotic resistance in *Helicobacter pylori* G27 and antibiotic-resistant derivatives. **(A)** Representative colony morphology of the parental *H. pylori* G27 strain, the levofloxacin-resistant derivative (Levo-R G27), and the metronidazole-resistant derivative (MTZ-R G27). **(B,C)** Representative growth of the parental and levofloxacin-resistant strains on agar plates containing increasing concentrations of levofloxacin, illustrating the increased MIC of the resistant derivative. **(D,E)** Representative growth of the parental and metronidazole-resistant strains on agar plates containing increasing concentrations of metronidazole, demonstrating growth of the resistant derivative at the highest concentration tested (32 μg/mL).

The parental *Helicobacter pylori* G27 strain exhibited minimum inhibitory concentration (MIC) values of 0.25 μg/mL for levofloxacin and 2 μg/mL for metronidazole (Figure 1 and Table 2). Following stepwise exposure to increasing antibiotic concentrations, resistant derivatives displaying markedly elevated MIC values were successfully generated. The levofloxacin-resistant derivative exhibited an MIC of 4 μg/mL, representing a 16-fold increase compared with the parental strain, whereas the metronidazole-resistant derivative remained viable at the highest concentration tested (32 μg/mL), indicating an MIC of >32 μg/mL (Figure 1 and Table 2). Interpreted against EUCAST clinical breakpoints (v14.0), the parental strain was susceptible to both agents, whereas the levofloxacin- and metronidazole-resistant derivatives exceeded the respective resistance breakpoints (levofloxacin, resistant >1 μg/mL; metronidazole, resistant >8 μg/mL). Representative colony growth on antibiotic-containing agar was consistent with the MIC results and confirmed the resistant phenotypes. These phenotypes remained unchanged after serial passaging in antibiotic-free medium, indicating stable maintenance of the acquired resistance.

**TABLE 2:** Minimum inhibitory concentrations (MICs) of levofloxacin and metronidazole against the parental and antibiotic-resistant *Helicobacter pylori* G27 strains.

Antibiotic	Concentration (μg/mL)	G27	Levo-R G27	MTZ-R G27
Levofloxacin	4.0	−	−	ND
	2.0	−	+	ND
	1.0	−	+	ND
	0.5	−	+	ND
	0.25	−	+	ND
	0.125	+	+	ND
	0.062	+	+	ND
	0.031	+	+	ND
Metronidazole	32	−	ND	+
	16	−	ND	+
	8	−	ND	+
	4	−	ND	+
	2	−	ND	+
	1	+	ND	+
	0.5	+	ND	+
	0.25	+	ND	+

MIC values were determined by agar dilution following incubation for 72 h under microaerophilic conditions. The MIC was defined as the lowest antibiotic concentration that completely inhibited visible bacterial growth. ND, not determined. + = visible growth; − = no visible growth; ND, not determined. Cross-resistance (i.e., susceptibility of the levofloxacin-resistant derivative to metronidazole, or of the metronidazole-resistant derivative to levofloxacin) was not tested, as strain generation targeted single-antibiotic selection; ND entries in the table reflect this scope rather than missing data.

MIC was defined as the lowest antibiotic concentration that completely inhibited visible bacterial growth following 72 h of incubation under microaerophilic conditions. Resistance was confirmed at the phenotypic level through MIC determination and stability testing after serial passaging on antibiotic-free medium; genotypic confirmation of resistance-associated mutations (e.g., rdxA/frxA for metronidazole, gyrA for levofloxacin) was not performed and is addressed as a limitation below.

### 3.2 Conservation of virulence gene profiles following antibiotic adaptation

PCR analysis was performed to assess the presence of 20 established *H. pylori* virulence-associated genes in the parental G27 strain and its antibiotic-resistant derivatives. All genes examined, including *cagA*, *vacA*, adhesin-encoding genes (*bab*, *alp*, *sab*, *hop*, *hpaA*), *ureA*, *ureB*, *ggt*, *dupA*, and *tolB*, were detected in all three strains, with identical amplification profiles (Table 3). No differences in gene presence or absence were observed between the parental and resistant derivatives.

**TABLE 3:** PCR-based virulence gene profiling of parental and antibiotic-resistant *Helicobacter pylori* G27 strains.

Gene	G27 (Parental)	Levofloxacin-resistant	Metronidazole-resistant
*ureA*	Present	Present	Present
*ureB*	Present	Present	Present
*cagA*	Present	Present	Present
*vacA* (s1/s2)	Present	Present	Present
*vacA* (m1/m2)	Present	Present	Present
*babA2*	Present	Present	Present
*babB*	Present	Present	Present
*babC*	Present	Present	Present
*alpA*	Present	Present	Present
*alpB*	Present	Present	Present
*sabA*	Present	Present	Present
*sabB*	Present	Present	Present
*hopZ*	Present	Present	Present
*hopQ*	Present	Present	Present
*oipA*	Present	Present	Present
*labA*	Present	Present	Present
*hpaA*	Present	Present	Present
*ggt*	Present	Present	Present
*dupA*	Present	Present	Present
*tolB*	Present	Present	Present

### 3.3 Morphological and biophysical characterization of outer membrane vesicles

**FIGURE 2:**
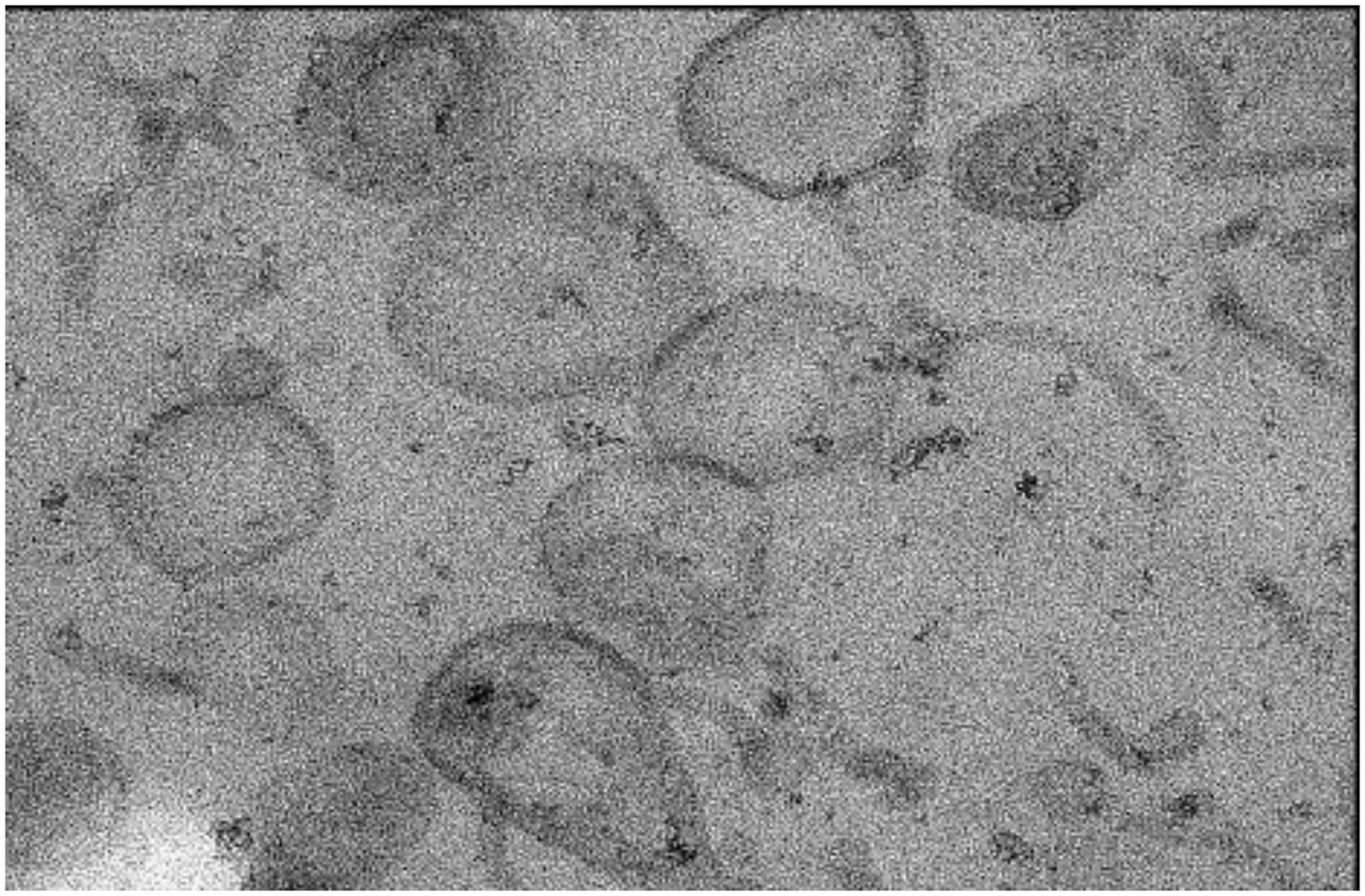
Transmission electron micrograph of outer membrane vesicles (OMVs) isolated from the parental *Helicobacter pylori* G27 strain. Representative TEM image showing spherical membrane-bound vesicles characteristic of *H. pylori* OMVs. Scale bar = 100 nm.

Transmission electron microscopy (TEM) of OMVs isolated from the parental *H. pylori* G27 strain revealed abundant spherical membrane-bound vesicles with morphologies consistent with previously described *H. pylori* OMVs (Figure 2).

**FIGURE 3:**
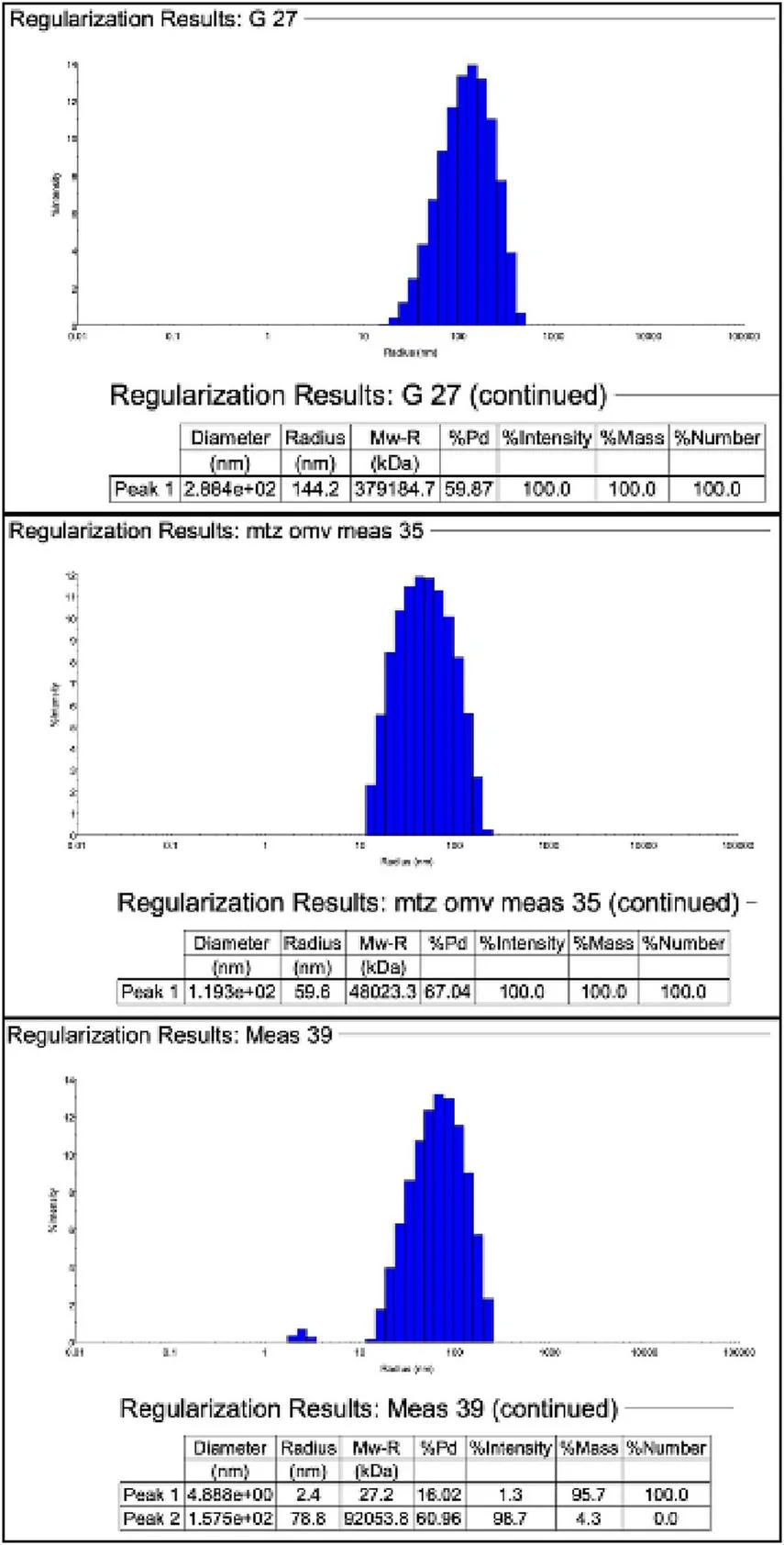
Dynamic light scattering (DLS) analysis of outer membrane vesicles derived from parental and antibiotic-resistant *Helicobacter pylori* G27 strains. Representative DLS size-distribution profiles of OMVs isolated from **(A)** parental G27, **(B)** MTZ-R G27, and **(C)** Levo-R G27 strains. Mean hydrodynamic diameter and polydispersity index (PDI) values are indicated for each preparation. Measurements represent single experimental determinations.

Dynamic light scattering (DLS) analysis demonstrated nanoscale vesicle populations in OMV preparations obtained from the parental and antibiotic-resistant strains (Figure 3 and Table 4). OMVs derived from the parental G27 strain exhibited a mean hydrodynamic diameter of 288.4 nm with a polydispersity index (PDI) of 0.60. In comparison, OMVs isolated from the metronidazole-resistant and levofloxacin-resistant derivatives displayed numerically smaller mean diameters of 119.3 nm (PDI = 0.67) and 157.5 nm (PDI = 0.61), respectively. Because DLS measurements were obtained from single experimental determinations, these values are presented as descriptive characterization only, without statistical comparison, and should not be interpreted as demonstrating a significant size difference between strains.

**TABLE 4:** Hydrodynamic diameter and polydispersity index (PDI) of OMVs isolated from parental and antibiotic-resistant *Helicobacter pylori* G27 strains.

Strain	Mean hydrodynamic diameter (nm)	PDI
G27	288.4	0.60
MTZ-R G27	119.3	0.67
Levo-R G27	157.5	0.61

Hydrodynamic diameter and polydispersity index (PDI) were determined by dynamic light scattering. Values represent single experimental measurements.

### 3.4 OMV protein content and SDS–PAGE profiling

**FIGURE 4:**
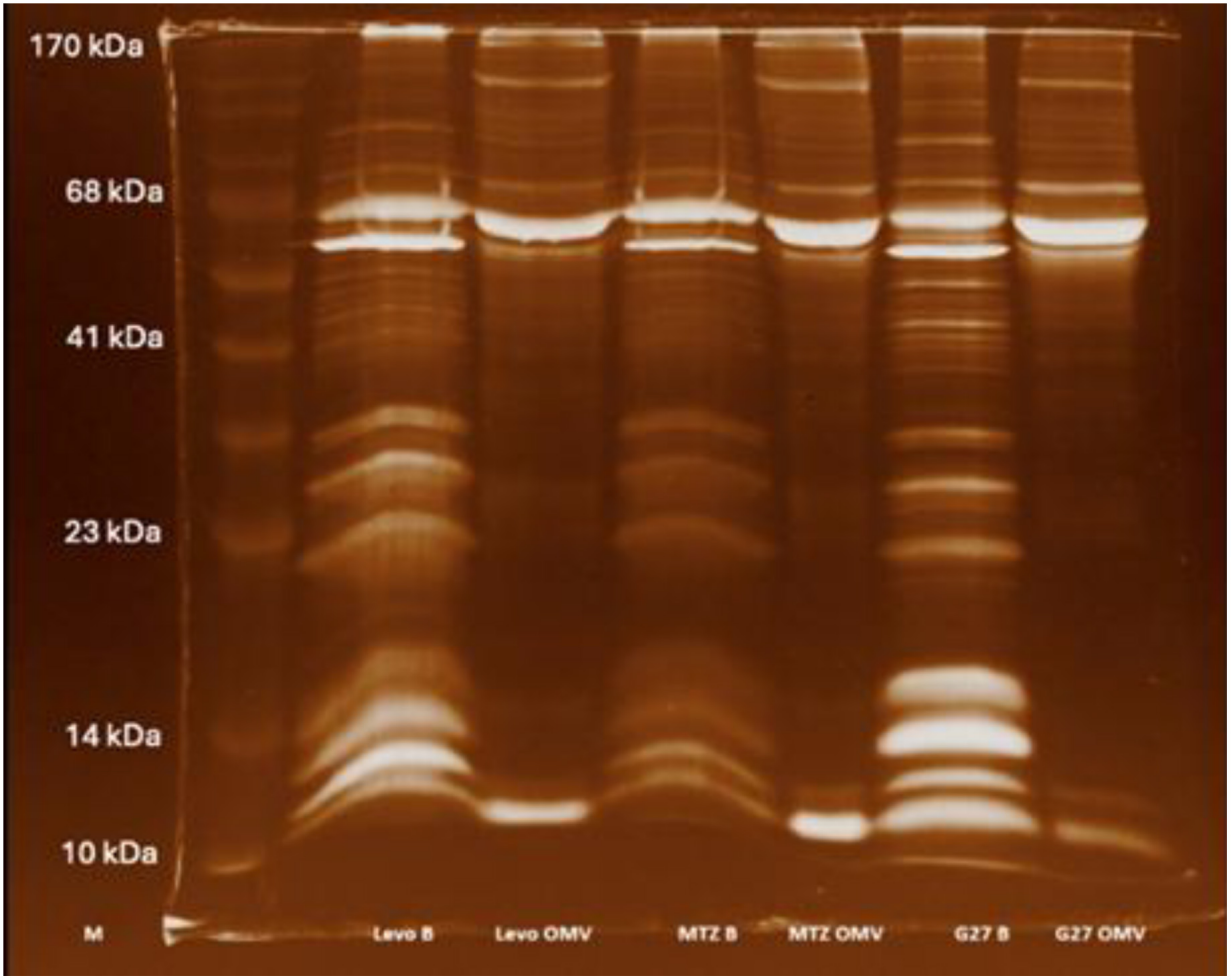
SDS–PAGE analysis of whole-cell and outer membrane vesicle (OMV)-associated proteins from parental and antibiotic-resistant *Helicobacter pylori* G27 strains. Whole-cell lysates and corresponding OMV protein fractions were separated by SDS–PAGE. Lane M, molecular weight marker; Levo B, whole-cell proteins of the levofloxacin-resistant strain; Levo OMV, OMV proteins of the levofloxacin-resistant strain; MTZ B, whole-cell proteins of the metronidazole-resistant strain; MTZ OMV, OMV proteins of the metronidazole-resistant strain; G27 B, whole-cell proteins of the parental G27 strain; G27 OMV, OMV proteins of the parental G27 strain.

SDS–PAGE analysis revealed distinct protein banding patterns between whole-cell lysates and the corresponding OMV fractions in the parental and antibiotic-resistant *H. pylori* G27 strains (Figure 4). Compared with whole-cell lysates, OMV preparations exhibited a more restricted protein profile, consistent with selective enrichment of a subset of bacterial proteins.

Quantification of OMV-associated proteins demonstrated strain-dependent differences in total protein content (Table 5). OMVs isolated from the parental G27 strain contained the highest protein concentration (3624.49 μg/mL), whereas those from the levofloxacin-resistant derivative exhibited the lowest protein concentration (1040.77 μg/mL). OMVs derived from the metronidazole-resistant strain showed an intermediate protein concentration (2070.64 μg/mL).

**TABLE 5:** Total protein concentration of OMVs isolated from parental and antibiotic-resistant *Helicobacter pylori* G27 strains.

Strain	OMV protein concentration (μg/mL)
Parental G27	3624.49
Levo-R G27	1040.77
MTZ-R G27	2070.64

### 3.5 LC–MS/MS reveals global proteomic remodeling following antibiotic resistance acquisition

LC–MS/MS-based proteomic profiling was performed to characterize the whole-cell proteomes of the parental *H. pylori* G27 strain and its levofloxacin- and metronidazole-resistant derivatives. Following quality filtering to remove low-confidence and non-reproducibly identified proteins, the resulting dataset was used for comparative proteomic and functional enrichment analyses.

**FIGURE 5:**
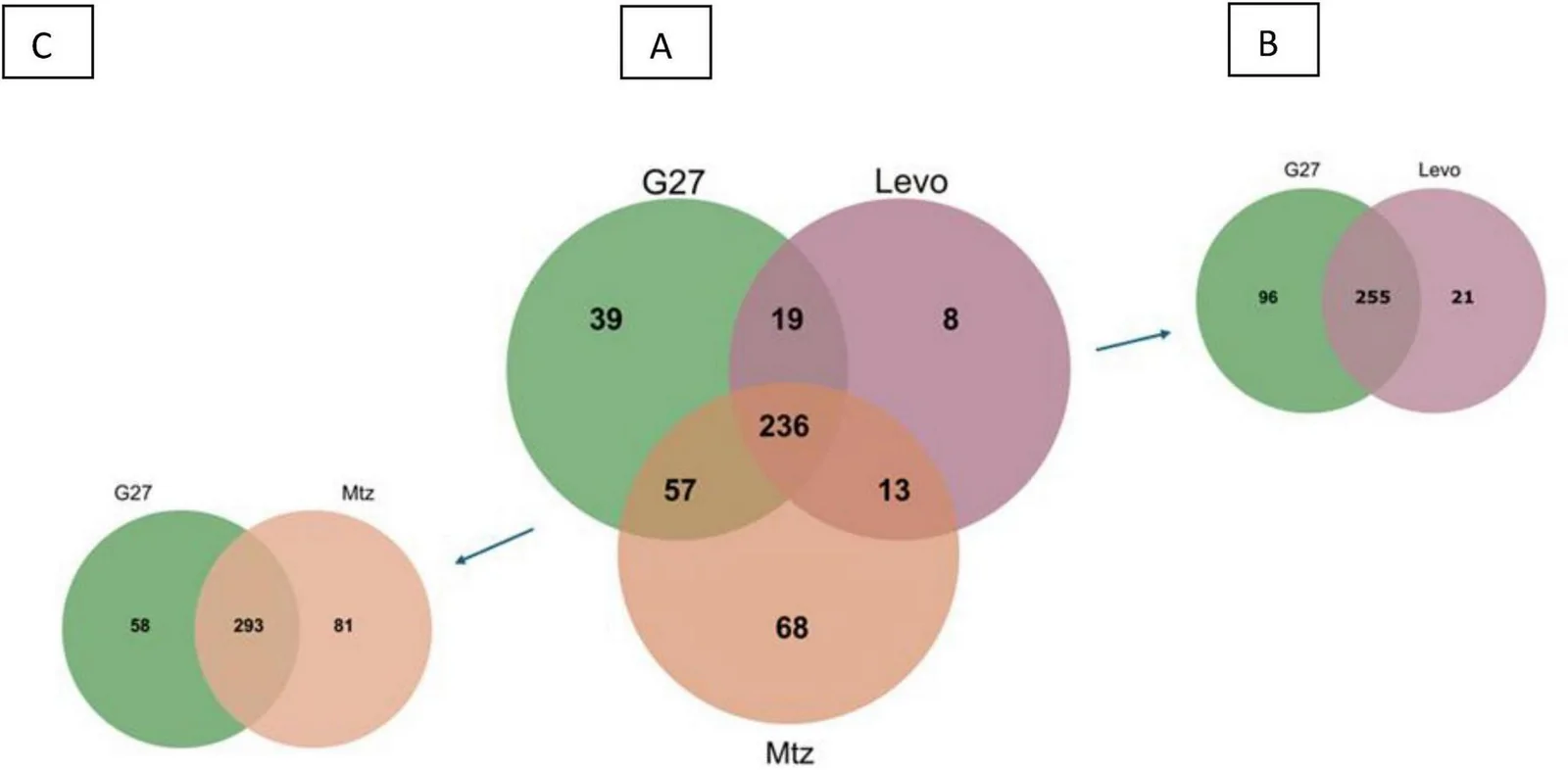
Comparative LC–MS/MS analysis of whole-cell proteomes from parental and antibiotic-resistant *Helicobacter pylori* G27 strains. **(A)** Three-way comparison of proteins identified in the parental G27 strain and its levofloxacin-resistant (Levo-R) and metronidazole-resistant (MTZ-R) derivatives. A total of 236 proteins were shared among all three strains, whereas 39, 8, and 68 proteins were uniquely detected in the parental, Levo-R, and MTZ-R strains, respectively. **(B)** Pairwise comparison of proteins identified in the parental G27 and Levo-R strains, showing 255 shared proteins together with proteins uniquely detected in each strain. **(C)** Pairwise comparison of proteins identified in the parental G27 and MTZ-R strains, illustrating the shared and strain-specific protein repertoires.

Comparative analysis, based on two independent biological replicates and interpreted qualitatively (detected/not detected; see section 2.10), identified a conserved core proteome comprising 236 proteins shared among all three strains (Figure 5A). In addition to this common protein repertoire, each strain exhibited a distinct set of uniquely detected proteins. The parental G27 strain contained 39 unique proteins, whereas the levofloxacin- and metronidazole-resistant derivatives contained 8 and 68 unique proteins, respectively (Figure 5A).

Pairwise comparisons further highlighted proteomic divergence associated with antibiotic resistance. The comparison between the parental and levofloxacin-resistant strains identified 255 shared proteins, together with 96 proteins detected exclusively in the parental strain and 21 proteins unique to the resistant derivative (Figure 5B). Likewise, comparison of the parental and metronidazole-resistant strains revealed 293 shared proteins, with 58 and 81 proteins uniquely detected in the parental and resistant strains, respectively (Figure 5C). Overall, these findings demonstrate that acquisition of antibiotic resistance is associated with apparent remodeling of the *H. pylori* cellular proteome while preserving a conserved core set of proteins.

### 3.6 Functional enrichment analysis of antibiotic resistance-associated whole-cell proteomes

**FIGURE 6:**
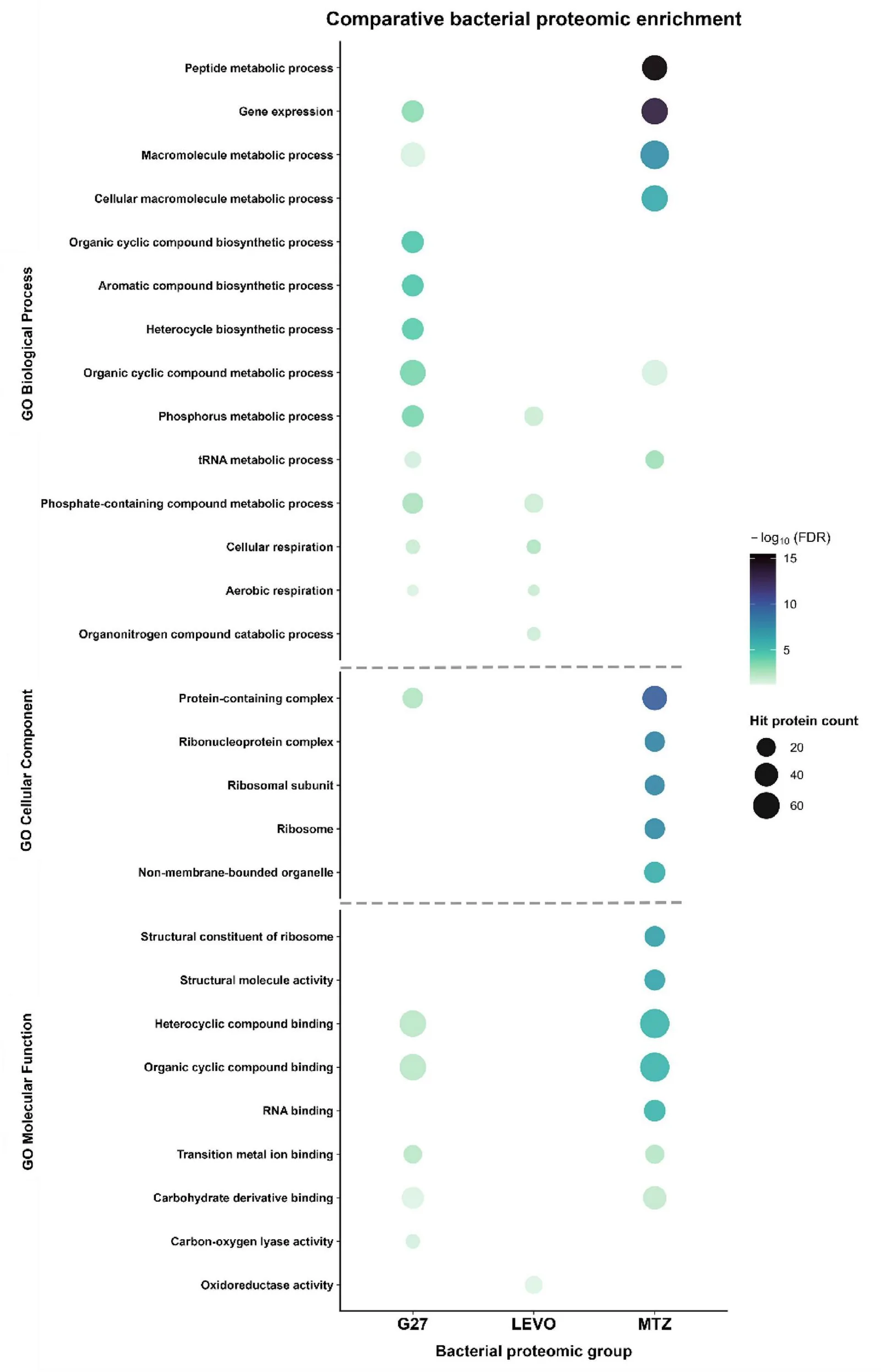
Comparative Gene Ontology enrichment analysis of strain-specific proteins identified in parental and antibiotic-resistant Helicobacter pylori G27 whole-cell proteomes. Bubble plots show enriched Gene Ontology (GO) terms associated with proteins uniquely detected in the parental G27 strain and its levofloxacin-resistant (Levo-R) and metronidazole-resistant (MTZ-R) derivatives. GO terms shared by all three groups were excluded to highlight strain- and resistance-associated functional patterns. Bubble size represents the number of proteins assigned to each enriched term, whereas color intensity indicates enrichment significance as - log10(FDR). Terms were filtered using FDR < 0.05 and at least two matched proteins, and the top enriched terms were selected within each group and GO category. This comparative visualization highlights distinct functional enrichment profiles among the parental and antibiotic-resistant proteomic states.

To investigate the biological significance of the strain-specific proteins identified by LC–MS/MS, Gene Ontology (GO) enrichment analysis was performed using proteins uniquely detected in the antibiotic-resistant derivatives. Comparative analysis revealed distinct functional signatures associated with levofloxacin and metronidazole resistance (Figure 6).

Proteins uniquely detected in the metronidazole-resistant strain were predominantly enriched in biological processes related to gene expression, translation, peptide biosynthesis, and ribosome-associated functions. In contrast, proteins unique to the levofloxacin-resistant strain were primarily associated with transmembrane transport, membrane organization, tRNA modification, and cellular responses to oxidative stress. Together, these findings indicate that the two resistance phenotypes are accompanied by distinct functional adaptations despite sharing a conserved core proteome.

**FIGURE 7:**
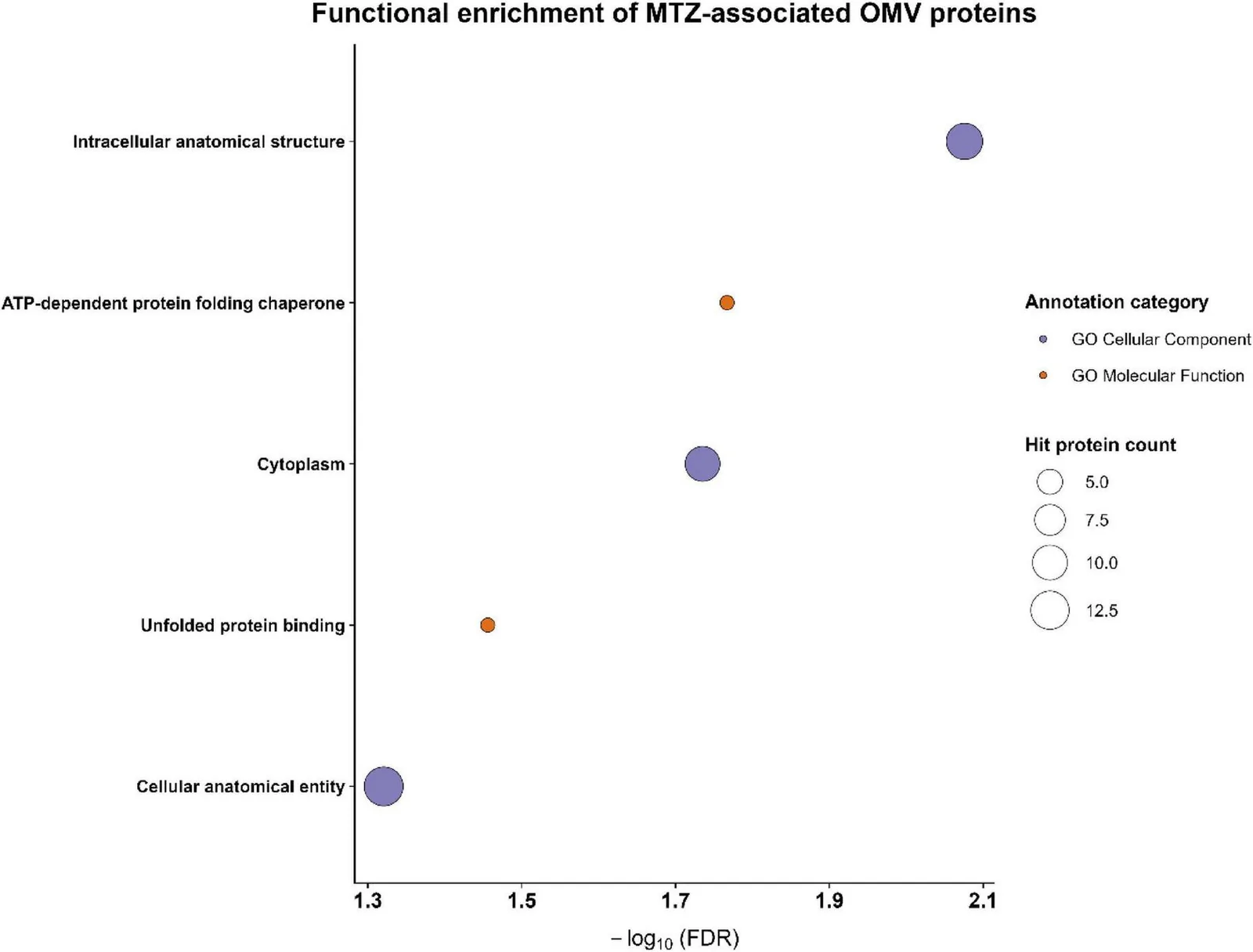
Functional enrichment profile of MTZ-associated OMV protein cargo. GO, KEGG, and Reactome enrichment analyses were performed on proteins identified in OMVs derived from the metronidazole-resistant H. pylori G27 derivative. Bubble size represents the number of matched OMV proteins, and the x-axis indicates enrichment significance as - log10(FDR). Colors indicate annotation categories. Terms were filtered at FDR < 0.05 with at least two matched OMV proteins. No significant enrichment was observed for parental G27- or Levo-R-derived OMV protein lists under the same thresholds.

To further characterize the biological pathways associated with antibiotic resistance, the strain-specific protein datasets were subjected to integrated GO, KEGG, and Reactome enrichment analyses (Figure 7). Functional enrichment demonstrated that resistance-associated proteins were linked to distinct metabolic, transport, and stress-response pathways, further supporting extensive remodeling of the *H. pylori* cellular proteome following acquisition of antibiotic resistance.

### 3.7 OMV proteomes show conserved core cargo and resistance-associated remodeling

**FIGURE 8:**
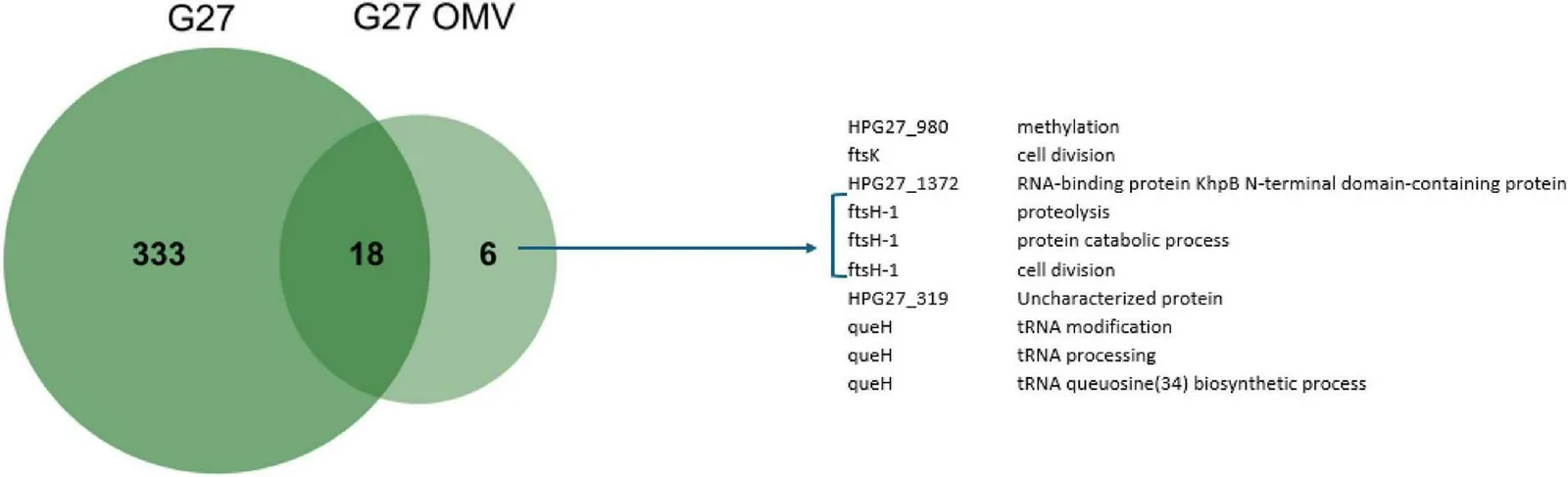
Comparative proteomic analysis of the *Helicobacter pylori* G27 strain and its outer membrane vesicles (OMVs).

**FIGURE 9:**
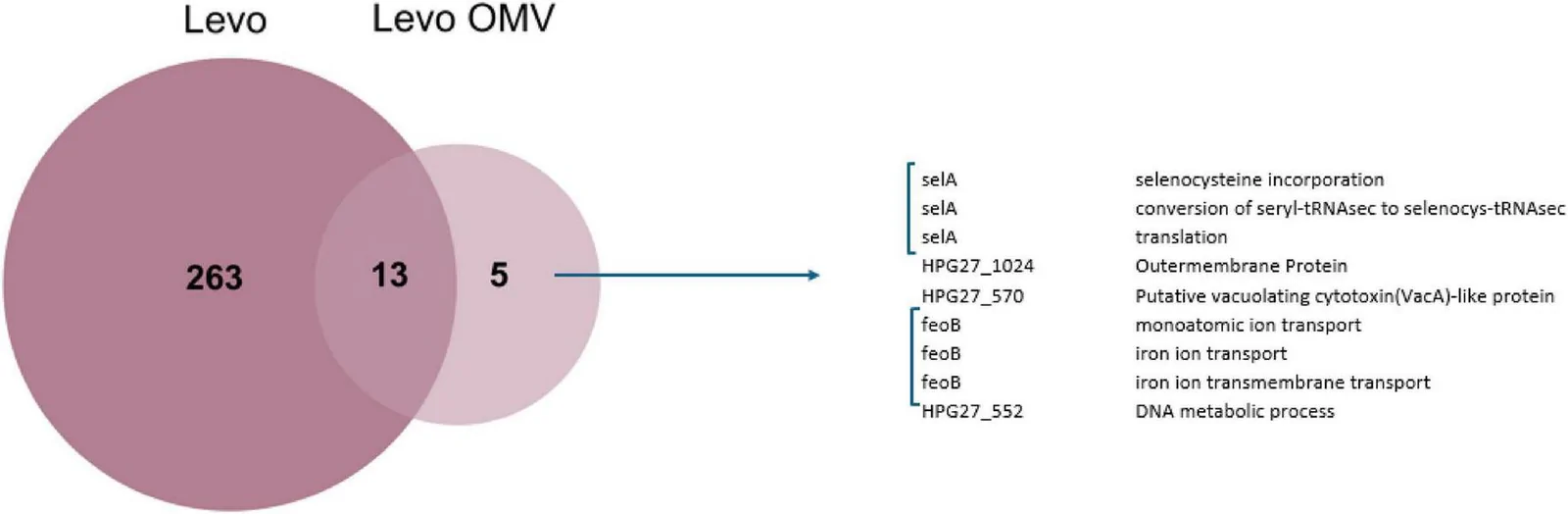
Comparative proteomic analysis of the levofloxacin-resistant *H. pylori* strain and its outer membrane vesicles (OMVs).

**FIGURE 10:**
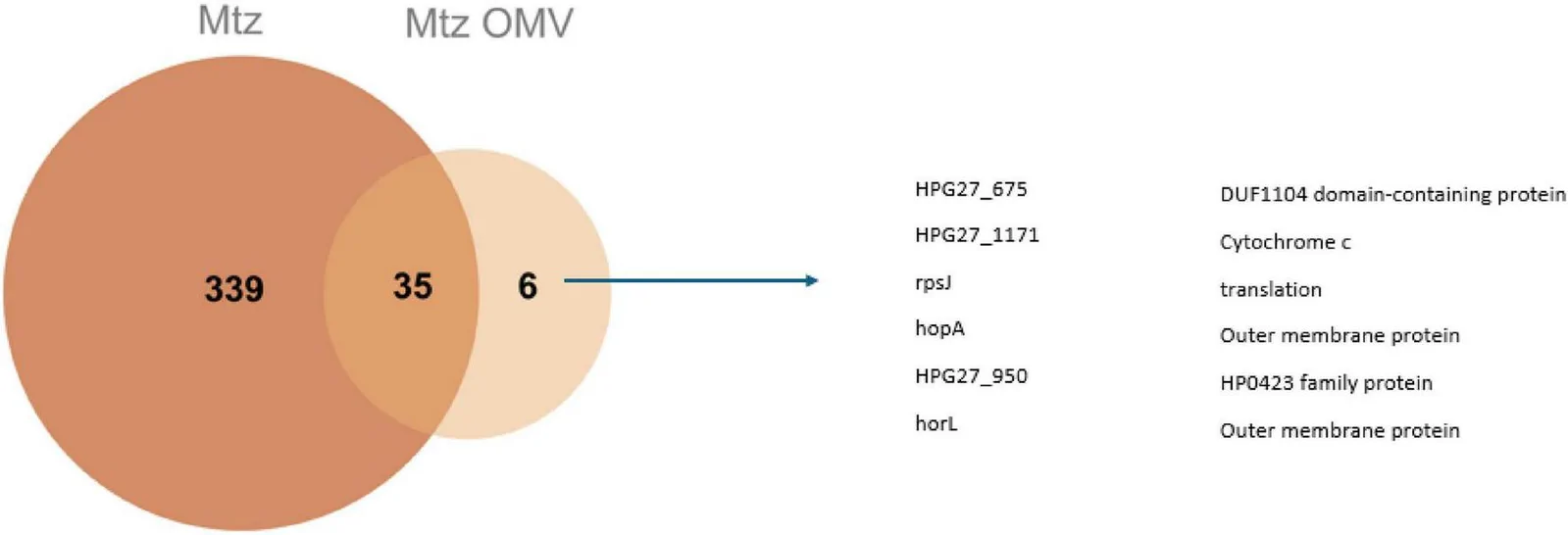
Comparative proteomic analysis of the metronidazole-resistant *H. pylori* strain and its outer membrane vesicles (OMVs).

LC–MS/MS analysis of outer membrane vesicles (OMVs) identified both conserved and strain-specific protein repertoires among the parental and antibiotic-resistant *H. pylori* G27 strains (Figures 8–10). Eight proteins were detected in OMVs from all three strains, representing a conserved vesicular protein core. In addition, 13 proteins were uniquely detected in OMVs derived from the parental G27 strain, whereas 6 and 28 proteins were unique to the levofloxacin- and metronidazole-resistant derivatives, respectively. A small number of proteins were also shared exclusively between pairs of strains, indicating partial overlap in OMV protein composition. Among the conserved OMV proteins identified in all three strains were the urease subunits UreA and UreB together with the molecular chaperones GroEL and GroES, indicating preservation of a core vesicle-associated protein repertoire despite resistance-associated remodeling. Overall, these findings demonstrate that antibiotic resistance is associated with alterations in OMV protein cargo while preserving a conserved subset of vesicle-associated proteins.

### 3.8 Functional remodeling of the OMV proteome

To investigate functional changes associated with altered OMV protein cargo, proteins identified in OMVs isolated from the parental and antibiotic-resistant *H. pylori* G27 strains were subjected to Gene Ontology (GO), KEGG pathway, and Reactome enrichment analyses (Figure 7). Comparative functional profiling revealed distinct enrichment patterns among OMVs derived from susceptible and resistant strains.

OMVs from the metronidazole-resistant strain showed enrichment of proteins associated with translation, ribosome-related functions, peptide biosynthesis, and cellular metabolic processes. In contrast, OMVs from the levofloxacin-resistant derivative were enriched for proteins involved in membrane-associated functions, transport, stress adaptation, and redox-related processes. KEGG and Reactome pathway analyses similarly revealed functional differences in vesicle-associated protein repertoires between the parental and resistant strains, indicating substantial remodeling of OMV protein cargo following acquisition of antibiotic resistance.

### 3.9 Selective protein packaging into outer membrane vesicles

To investigate selective protein packaging into outer membrane vesicles (OMVs), whole-cell proteomes were compared with their corresponding OMV proteomes for each strain (Figures 8–10). Although most proteins were shared between whole-cell and OMV preparations, each OMV proteome also contained proteins that were not detected in the corresponding whole-cell proteome.

In the parental G27 strain, six proteins were detected exclusively in OMVs, including FtsH-1 and QueH (Figure 8). Similarly, OMVs derived from the levofloxacin-resistant strain contained five OMV-specific proteins, including SelA and FeoB (Figure 9). In the metronidazole-resistant strain, six proteins were identified exclusively in OMVs, including RpsJ, HopA, and HorL (Figure 10). Collectively, these findings are consistent with selective enrichment of specific proteins during OMV biogenesis; however, because whole-cell and OMV samples were prepared and analyzed separately, differences in sample preparation or digestion efficiency between the two fractions cannot be excluded as a contributing factor, and this pattern should not be taken as definitive proof of active, regulated packaging rather than passive incorporation of cellular proteins.

### 3.10 Differential representation of virulence- and stress-associated proteins in OMVs

Proteomic profiling further revealed differential representation of virulence- and stress-associated proteins among OMVs derived from susceptible and antibiotic-resistant strains (Table 1). CagA was detected in OMVs from the parental strain but was not identified in OMVs from either resistant derivative, despite being present in the corresponding whole-cell proteomes. In contrast, VacA was detected in OMVs from both resistant strains but was absent from OMVs derived from the parental strain.

OMVs from the levofloxacin-resistant strain additionally contained proteins associated with stress adaptation and redox homeostasis, including SelA, together with the putative drug-response protein HPG27_591. In the metronidazole-resistant strain, the outer membrane proteins HopA and HorL were specifically detected in both whole-cell and OMV proteomes. These findings demonstrate that antibiotic resistance is accompanied by changes in the representation of virulence-, membrane-, and stress-associated proteins within OMVs.

## 4 Discussion

The increasing prevalence of antibiotic-resistant *Helicobacter pylori* continues to compromise the effectiveness of eradication therapy and highlights the need to better understand the physiological adaptations accompanying the development of antimicrobial resistance. Although resistance in *H. pylori* is primarily associated with mutations in antibiotic target genes, accumulating evidence indicates that successful adaptation also involves broader changes in bacterial physiology, including metabolic reprogramming, membrane remodeling, and stress-response mechanisms. In the present study, metronidazole- and levofloxacin-resistant derivatives of the *H. pylori* G27 strain were generated through stepwise antibiotic selection and comparatively characterized using LC–MS/MS analysis of both whole-cell lysates and outer membrane vesicles (OMVs). By integrating cellular and vesicular proteomes, this study provides insight into proteomic changes associated with antibiotic adaptation and demonstrates that resistance-associated remodeling extends beyond the bacterial cell to include alterations in OMV protein composition.

Comparative analysis showed that the majority of proteins were shared between the parental strain and its resistant derivatives, indicating preservation of a conserved cellular proteome despite prolonged antibiotic selection. At the same time, each resistant derivative contained a distinct subset of proteins that was not detected in the susceptible strain. Functional enrichment analyses indicated that these proteins were associated primarily with translation, membrane-associated functions, transport processes, metabolic adaptation, and cellular stress responses. Similar physiological adjustments have been described in bacteria exposed to antibiotic stress, where coordinated regulation of protein synthesis, membrane homeostasis, and energy metabolism contributes to bacterial survival under adverse conditions (Kulp and Kuehn, 2010; Olofsson et al., 2010). Rather than representing isolated changes associated with antibiotic targets, the present findings support the concept that acquisition of resistance is accompanied by broader proteomic reorganization affecting multiple cellular pathways.

Among the proteins identified in the resistant strains, HefB is of particular interest because resistance–nodulation–division (RND) efflux systems have been implicated in multidrug resistance in *H. pylori*. Increased efflux activity reduces intracellular antibiotic accumulation and may complement target-site mutations, including alterations in *gyrA* and *gyrB*, which are well-established determinants of fluoroquinolone resistance (Parker et al., 2010; Tegtmeyer et al., 2016). Efflux activity and hefB expression were not directly measured in the present study, so the detection of HefB in both antibiotic-resistant derivatives, but not in the parental strain, remains a correlative and, at this stage, speculative observation; it is nonetheless consistent with the enrichment of membrane-associated and transport-related functional categories observed in the corresponding proteomes. Direct functional validation, such as efflux pump activity or inhibition assays, will be required before membrane transport systems can be concluded to contribute to fluoroquinolone resistance adaptation in this strain.

Proteomic analysis further demonstrated that resistance-associated remodeling was not confined to the cellular proteome but also involved changes in OMV protein composition. Outer membrane vesicles are increasingly recognized as active components of bacterial physiology that contribute to environmental adaptation, intercellular communication, and interactions with host tissues through the transport of proteins, lipids, and nucleic acids (Amieva and Peek, 2016; Backert et al., 2010; Bonnington and Kuehn, 2014; Celli and Tsolis, 2015). In the present study, OMVs from all three strains shared a conserved subset of proteins, including the urease subunits UreA and UreB, the molecular chaperones GroEL and GroES, and the adhesin HopQ. Preservation of these proteins across susceptible and resistant strains suggests the existence of a conserved vesicle-associated protein repertoire that is maintained despite resistance-associated remodeling of other OMV components. Given the established roles of urease in gastric colonization and GroEL/GroES in stress tolerance, their consistent detection within OMVs is compatible with the contribution of vesicles to bacterial survival under both gastric and antibiotic-associated stress conditions (Yonezawa et al., 2015; Zhang et al., 2020).

A subsequent observation was that resistance-associated remodeling affected not only the overall composition of OMVs but also the representation of proteins involved in virulence and stress adaptation. Although CagA was detected in the whole-cell proteomes of all three strains, it was identified in OMVs only from the parental strain, whereas VacA was detected in OMVs from both resistant derivatives. Because both proteins remained detectable within the corresponding whole-cell proteomes, these differences are unlikely to reflect changes in protein expression alone. Instead, they suggest that acquisition of antibiotic resistance may influence the selective incorporation of virulence-associated proteins into OMVs. Previous studies have established that OMVs serve as important vehicles for the delivery of CagA, VacA, and other bacterial effectors to host cells, thereby contributing to immune modulation and persistence during *H. pylori* infection (Ellis and Kuehn, 2010; Olofsson et al., 2010; Schwechheimer and Kuehn, 2015; Turner et al., 2018. The differential representation of these virulence factors observed in the present study therefore raises the possibility that antibiotic adaptation may influence not only bacterial survival but also the composition of vesicle-associated virulence determinants.

Comparison of whole-cell and corresponding OMV proteomes is consistent with, though does not by itself prove, selective protein packaging during vesicle biogenesis. Distinct protein packaging behaviors were observed across the studied strains. FtsH-1 and QueH in the G27 strain, FeoB in the LEVO-resistant derivative, and HopA in the MTZ-resistant derivative were detected exclusively in OMV fractions, with no corresponding identification in whole-cell lysates. In contrast, HorL was detected in LEVO whole-cell lysates and MTZ OMVs, while SelA was present in LEVO OMVs and MTZ whole-cell samples. Although the absence of these proteins from whole-cell datasets may partly reflect the analytical detection limits of LC–MS/MS, their consistent detection within OMVs is compatible with selective enrichment during vesicle formation rather than passive incorporation of cellular contents. Many of these proteins are associated with protein quality control, redox homeostasis, iron acquisition, or outer membrane organization, processes that are closely linked to bacterial adaptation under environmental and antibiotic-induced stress (Blair et al., 2015; Dwyer et al., 2014; Olofsson et al., 2010; Turner et al., 2018. Collectively, these observations support the view that OMVs represent a regulated extracellular compartment whose protein composition reflects the physiological state of the bacterium rather than a random sample of the cellular proteome. Taken together, these findings suggest that remodeling of OMV cargo represents an additional adaptive layer accompanying antibiotic resistance in *H. pylori*, potentially influencing bacterial survival, interactions with the host, and persistence under antimicrobial pressure.

### 4.1 Study limitations

As an exploratory, hypothesis-generating study, several limitations warrant discussion. The proteomic comparisons rely on two biological replicates and qualitative detected/not-detected calls rather than quantitative label-free abundance data, and the gel-based digestion workflow further constrains proteome depth. Proteins identified in only one strain, including the differential representation of CagA and VacA, should therefore be interpreted with caution, since exclusive detection can reflect the sensitivity limits of the LC–MS/MS workflow as much as genuine biological specificity. Orthogonal validation (Western blotting, targeted PRM-MS) and quantitative label-free or TMT-based analyses with additional replicates will be needed before these differences can be considered robust.

A related caveat concerns strain history: resistant derivatives were generated under sustained antibiotic exposure, so some of the observed proteomic changes may reflect adaptation to that exposure rather than the resistance phenotype *per se*. Canonical resistance determinants (*gyrA, rdxA, frxA*) were not confirmed by sequencing in this work, so the proteomic remodeling described here cannot yet be attributed to specific genetic mechanisms; pairing genomic characterization with comparative proteomics is a natural next step toward defining genotype–proteotype relationships in resistant *H. pylori*.

The derivatives all originate from a single G27 background, and cross-resistance between the metronidazole- and levofloxacin-resistant lines was not tested, as each was only characterized against its selecting antibiotic. Extending this work to a broader panel of clinical isolates with diverse genetic backgrounds will be important for assessing how generalizable these findings are. Dynamic light scattering was performed once per OMV preparation and is reported descriptively rather than statistically. Vesicles were purified by differential ultracentrifugation alone, without a density-gradient step or verification of purity using cytoplasmic markers such as EF-Tu; low-level contamination from co-sedimenting membrane fragments or cytoplasmic proteins therefore cannot be excluded, and marker-based purity assessment should be incorporated in future preparations.

Finally, this study is descriptive at the proteomic level and does not address whether the cargo changes observed translate into altered biological activity. The roles proposed here for resistance-associated OMV proteins in host-cell interaction, immune modulation, stress adaptation, or antibiotic tolerance remain hypotheses. Testing them will require functional work, for example gastric epithelial infection models, OMV-mediated antibiotic-protection assays, and targeted validation of candidates such as the efflux-associated protein HefB. Peptide-spectrum match and unique peptide counts for all identified proteins are provided in Supplementary Table S_PSM to support confidence in protein identification; given the qualitative design of this study, these values should not be read as a proxy for relative abundance.

Taken together, despite these limitations, the consistent compositional differences observed between parental and resistant OMVs offer an initial framework for how antibiotic resistance reshapes the *H. pylori* vesicle proteome, and provide a basis for the quantitative and functional studies needed to build on it.

## Data Availability

The original contributions presented in the study are publicly available. This data can be found in the ProteomeXchange Consortium via the MassIVE repository under accession number PXD083327 (MassIVE accession: MSV000103038).
