# Supplementary material for: Proteomic remodeling of outer membrane vesicles in antibiotic-resistant Helicobacter pylori reveals adaptive stress responses
Source: Front Microbiol. 2026 Sep 4;17:1944144. doi: 10.3389/fmicb.2026.1944144 (PMC13586027; doi:10.3389/fmicb.2026.1944144)
Supplement: Supplementary file 1 [file Table_1.docx]

**Supplementary Fig. S1. Combined OMV protein mapping to bacterial GO terms across G27, LEVO and MTZ.**

**Supplementary Fig. S1.** Combined OMV protein mapping to bacterial GO terms across G27, LEVO and MTZ. The combined chord diagram highlights group-level differences in OMV protein-GO mapping. MTZ-associated OMV proteins contribute the densest set of ribbons, whereas G27 and LEVO show comparatively narrower mapping patterns. Ribbons represent OMV protein-to-GO term membership; GO term sectors are shown on the left side of the chord plots, and OMV protein sectors are shown on the right.

**Supplementary Fig. S2. G27 OMV proteins mapped to G27 bacterial GO terms.**

**Supplementary Fig. S2.** G27 OMV proteins mapped to G27 bacterial GO terms. G27 OMV proteins mainly map to broad core terms, including metabolic process, cellular process, cytoplasm/intracellular structure, catalytic activity and binding. Ribbons represent OMV protein-to-GO term membership; GO term sectors are shown on the left side of the chord plots, and OMV protein sectors are shown on the right. .

**Supplementary Fig. S3. LEVO OMV proteins mapped to LEVO bacterial GO terms.**

**Supplementary Fig. S3.** LEVO OMV proteins mapped to LEVO bacterial GO terms. LEVO OMV proteins show a compact mapping pattern dominated by core metabolic/cellular GO terms and a limited number of OMV protein nodes. Ribbons represent OMV protein-to-GO term membership; GO term sectors are shown on the left side of the chord plots, and OMV protein sectors are shown on the right.

**Supplementary Fig. S4. MTZ OMV proteins mapped to MTZ bacterial GO terms.**

**Supplementary Fig. S4.** MTZ OMV proteins mapped to MTZ bacterial GO terms. MTZ OMV proteins show the broadest mapping pattern, with multiple proteins connected to cellular/metabolic functions and stress- or housekeeping-associated modules. Ribbons represent OMV protein-to-GO term membership; GO term sectors are shown on the left side of the chord plots, and OMV protein sectors are shown on the right.

- Koymak istersek makale metnine entegre edebileceğimiz metin

To further contextualize the functional relevance of OMV protein cargo, OMV-associated proteins were mapped onto GO terms derived from the corresponding bacterial proteomic enrichment analyses and visualized using chord diagrams (**Supplementary File 1; Supplementary Figures S1–S4**). This analysis showed that OMV proteins were mainly connected to core bacterial cellular and metabolic modules, including cellular process, metabolic process, nitrogen compound metabolism, catalytic activity, binding, and cytoplasmic/intracellular structural terms. Although G27 and LEVO OMVs displayed relatively restricted connectivity, MTZ-associated OMVs exhibited a broader and denser protein–GO network, involving proteins related to protein folding/chaperone function, DNA replication or repair, translation, and central metabolism. These observations suggest that MTZ exposure may promote a condition-specific remodeling of OMV protein cargo toward stress-adaptive and housekeeping functions. Given that the chord diagrams represent qualitative protein–term mapping rather than direct evidence of pathway activation, these findings should be interpreted as hypothesis-generating and warrant targeted validation of candidate OMV cargo proteins.
